# A case-based approach to implementing guidelines for stroke prevention in patients with atrial fibrillation: balancing the risks and benefits

**DOI:** 10.1186/s12959-015-0056-y

**Published:** 2015-08-21

**Authors:** Alpesh Amin, Steven Deitelzweig

**Affiliations:** Department of Medicine, Executive Director, Hospitalist Program, University of California, UCIMC, 101 The City Drive South, Building 26, Room 1005, ZC-4076H, Mail Code: 4076, Irvine, CA 92868 USA; University of Queensland, System Chairman of Hospital Medicine, Medical Director for Regional Business Development, Ochsner Health System, New Orleans, LA 70112 USA

**Keywords:** Risk, Benefit, Guidelines, Stroke, Atrial fibrillation

## Abstract

Atrial fibrillation (AF) puts patients at risk of complications, including stroke. Warfarin therapy has been the mainstay of antithrombotic treatment for reducing the risk of stroke in AF. However, warfarin has limitations that have motivated development of several novel oral anticoagulants (NOACs), including dabigatran, rivaroxaban, apixaban, and edoxaban. Clinical trials demonstrate that the NOACs offer efficacy and safety that are equivalent to, or better than, those of warfarin for reducing the risk of stroke in patients with nonvalvular AF. This review examines stroke risk reduction in patients with AF from the perspective of the clinician balancing the risks and benefits of treatment options, evaluates the most recent guidelines, and discusses 2 hypothetical patient cases to better illustrate how clinicians may apply available data in the clinical setting. We reviewed guidelines for the reduction of stroke risk in AF and data from clinical trials on the NOACs. Choosing antithrombotic treatment involves assessing the benefits of therapy versus its risks. Risk indexes, including CHADS_2_, CHA_2_DS_2_-VASc, and HAS-BLED can help determine how to treat patients with AF. Current guidelines suggest using these risk indexes to customize treatment to individual patients. Many current treatment guidelines also incorporate recommendations for the use of NOACs as an alternative to warfarin. As additional data emerge and guidelines are updated, these recommendations will likely evolve. In the interim, clinicians may consider published guidelines and clinical trial results on NOACs. Real-world experience will provide clinicians with additional insight into their treatment decisions.

## Introduction

Successful healthcare interventions improve patient outcomes and reduce costs associated with disease management. Guidelines provide a standardized, evidence-based approach to the diagnosis and treatment of disease, with a goal of optimizing health outcomes. When available, preventive measures to reduce the risk of disease complications are an important element of treatment. Atrial fibrillation (AF), which affected an estimated 5.2 million adults in the United States in 2010, is a disease state in which patients are at risk of complications, including stroke [1]. AF is a highly prevalent cardiac arrhythmia that imparts a 2- to 7-times greater risk of embolic or transient stroke [2, 3]. AF-related stroke is more severe than stroke without AF, and is associated with greater disability and subsequent medical needs [4, 5]. The high AF-related morbidity is reflected in a substantial economic burden. For 2010, the projected incremental cost of AF in the United States ranged from $6.0 billion to $26.0 billion [6].

Antithrombotic therapy can reduce the risk of stroke in patients with AF [7–11]; however, many at-risk patients are untreated or undertreated [12–15]. Antithrombotic treatment for stroke risk reduction in patients with AF has traditionally focused on anticoagulation with a vitamin K antagonist (VKA), primarily warfarin [2]. Warfarin is highly effective for reducing the risk of stroke. However, warfarin has a narrow therapeutic window and many patients have difficulty remaining within the therapeutic range. Under-anticoagulation may lead to stroke, whereas over-anticoagulation may result in bleeding. Warfarin can be challenging to use in the clinical setting due to dietary and drug interactions, and variability in dose response due to genetic and environmental factors [13]. Warfarin necessitates regular monitoring and dose adjustments to enable individual patients to receive safe and effective treatment [13, 16]. Many physicians overestimate bleeding risk and underestimate the benefit of stroke prevention [17]. Bleeding risk and concerns about bleeding risk, as well as other clinical features of warfarin, may lead clinicians to rule it out as a treatment option; hence, many patients remain untreated [18–20]. Until recently, aspirin was the only treatment alternative for patients unsuitable for VKA therapy. Although bleeding risk is lower and administration is simpler with aspirin, the efficacy of aspirin in stroke prevention is inferior to that of warfarin [8, 21]. Thus, the conventional treatment options of warfarin and aspirin have left a segment of the patient population untreated and/or undertreated and at increased risk of stroke [22].

The limitations of the traditional options for stroke prevention in patients with AF have motivated the development of several novel oral anticoagulants (NOACs). Recent clinical trials have found that the NOACs offer efficacy and safety equivalent to, or better than, those of warfarin for reducing the risk of stroke in patients with nonvalvular AF (NVAF) [23–26]. As more clinical experience is gained with the NOACs, changes in treatment paradigms and guidelines will undoubtedly ensue. This review examines stroke prevention in patients with AF from the perspective of the clinician trying to balance the risks and benefits of treatment options. Use of treatment guidelines and the application of findings from recent clinical trials will help facilitate high-quality and cost-effective medical care; however, effective care must be tailored to the individual patient. In addition to reviewing the most recent guidelines for stroke prevention in AF, we will discuss 2 hypothetical patient cases to better understand how clinicians may apply these measures and the available data in the clinical setting.

### Stroke risk reduction in NVAF with NOACs

Several NOACs are available in the United States for reducing the risk of stroke in patients with NVAF, including the direct thrombin inhibitor dabigatran and the factor Xa inhibitors rivaroxaban, apixaban and edoxaban [26–30]. A brief summary of the results of key phase 3 clinical trials comparing the NOACs with either warfarin or aspirin for reducing the risk of stroke in NVAF is provided in Table 1 [23–26, 31, 32]. These results provide support for the integration of NOACs into treatment paradigms for stroke risk reduction in AF as potential first-line options.

**Table 1:** Phase 3 clinical trials of NOACs: study design and outcomes

Trial name	Trial design	No. of patients	Outcomes (annual rate vs. comparator,^a^ %/y)			
			Stroke/Systemic embolism	All-cause mortality^b^	Major bleeding^b^	Intracranial hemorrhage^b^
RE-LY^c^ (dabigatran) [23, 31]	PROBE design^d^	*N* = 18113 Dabigatran 150 mg bid: *n* = 6076	Dabigatran 150 mg: RR 0.66 (95 % CI 0.53, 0.82; *p* < 0.001 for superiority)	Dabigatran 150 mg: RR 0.88 (95 % CI 0.77, 1.00; *p* = 0.051)	Major bleeding: Dabigatran 150 mg: RR 0.93 (95 % CI 0.81, 1.07; *p* = 0.31)	Dabigatran 150 mg: RR 0.40 (95 % CI 0.27, 0.60; *p* < 0.001)
		Dabigatran 110 mg bid: *n* = 6015	Dabigatran 110 mg: RR 0.91 (95 % CI 0.74, 1.11; *p* < 0.001 for noninferiority)	Dabigatran 110 mg: RR 0.91 (95 % CI 0.80, 1.03; *p* = 0.13)	Dabigatran 110 mg: RR 0.80 (95 % CI 0.69, 0.93; *p* = 0.003)	Dabigatran 110 mg: RR 0.31 (95 % CI 0.20, 0.47; *p* < 0.001)
		Warfarin (adjusted dose, target INR 2.0–3.0): *n* = 6022				
ROCKET-AF (rivaroxaban) [25]	Randomized, double-blind, double-dummy, noninferiority trial	*N* = 14264 Rivaroxaban 20 mg/d: *n* = 7131		HR 0.85 (95 % CI 0.70, 1.02; *p* = 0.07)	Major and CRNM bleeding: HR 1.03 (95 % CI 0.96, 1.11; *p* = 0.44)	HR 0.67 (95 % CI 0.47, 0.93; *p* = 0.02)
		Warfarin (adjusted dose, target INR 2.0–3.0): *n* = 7133	HR 0.88 (95 % CI 0.75, 1.03; *p* < 0.001 for noninferiority; *p* = 0.12 for superiority)^e^		Major bleeding: HR 1.04 (95 % CI 0.90, 1.20; *p* = 0.58)	
ENGAGE AF-TIMI 48 (edoxaban) [26]	Randomized, double-blind, double-dummy, noninferiority trial	*N* = 21105 Edoxaban 60 mg once daily: *n* = 7035	Edoxaban 60 mg: HR 0.79 (97.5 % CI 0.63, 0.99; *p* < 0.001 for noninferiority; *p* = 0.08 for superiority)^f^	Edoxaban 60 mg: HR, 0.92 (95 % CI. 0.83, 1.01; *p* = 0.08)	Major bleeding: Edoxaban 60 mg: HR 0.80 (95 % CI 0.71, 0.91; *p* < 0.001)	Edoxaban 60 mg: HR 0.47 (95 % CI 0.34, 0.63; *p* < 0.001)
		Edoxaban 30 mg once daily: *n* = 7034	Edoxaban 30 mg: HR 1.07 (97.5 % CI 0.87, 1.31; *p* = 0.005 for noninferiority; *p* = 0.10 for superiority)^f^	Edoxaban 30 mg: HR, 0.87 (95 % CI, 0.79, 0.96; *p* = 0.006)	Edoxaban 30 mg: HR 0.47 (95 % CI 0.41, 0.55; *p* < 0.001)	Edoxaban 30 mg: HR 0.30 (95 % CI 0.21, 0.43; *p* < 0.001)
		Warfarin (adjusted dose, target INR 2.0–3.0): *n* = 7036				
ARISTOTLE (apixaban) [24]	Randomized, double-blind, double-dummy, noninferiority trial	*N* = 18201 Apixaban 5 mg bid: *n* = 9120	HR 0.79 (95 % CI 0.66, 0.95; *p* < 0.001 for noninferiority; *p* = 0.01 for superiority)	HR 0.89 (95 % CI 0.80, 0.998; *p* = 0.047)	Major bleeding: HR 0.69 (95 % CI 0.60, 0.80; *p* < 0.001)	HR, 0.42 (95 % CI, 0.30, 0.58; *p* < 0.001)
		Warfarin (adjusted dose, target INR 2.0–3.0): *n* = 9081				
AVERROES (apixaban) [32]	Randomized, double-blind, double-dummy, superiority trial^g^	*N* = 5599 Apixaban 5 mg bid: *n* = 2808	HR 0.45 (95 % CI 0.32, 0.62; *p* < 0.001)	HR 0.79 (95 % CI 0.62, 1.02; *p* = 0.07)	Major bleeding: HR 1.13 (95 % CI 0.74, 1.75; *p* = 0.57)	HR 0.85 (95 % CI 0.38, 1.90; *p* = 0.69)
		Aspirin 81–324 mg/d: *n* = 279				

*bid* twice daily, *CI* confidence interval, *CRNM* clinically relevant nonmajor, *HR* hazard ratio, *INR* international normalized ratio, *ITT* intention-to-treat, *NOAC* novel oral anticoagulant, *PROBE* prospective, randomized, open, blinded end-point, *RR* relative risk

^a^NOACs were compared with warfarin in the RE-LY, ROCKET-AF, ENGAGE AF-TIMI 48 and ARISTOTLE trials and with aspirin in the AVERROES trial

^b^p values are for superiority

^c^Values for RE-LY are given as RR

^d^PROBE design

^e^ITT population

^f^Modified ITT (mITT) population

^g^This study was terminated early due to treatment benefit in favor of apixaban

### Guidelines and quality measures for stroke risk reduction in AF

Several guidelines discuss stroke risk reduction in patients with AF (Table 2), including recommendations from the American College of Cardiology (ACC), the American College of Chest Physicians (ACCP), the American Heart Association (AHA), the American Stroke Association (ASA), the Heart Rhythm Society (HRS), the European Society of Cardiology (ESC), and the American Academy of Neurology (AAN) [7–11]. In general, treatment decisions are based on an assessment of the individual patient’s stroke risk, with a concurrent assessment of bleeding risk and other patient-related factors, such as his or her ability to adhere to monitoring requirements and personal preferences [7, 10]. However, specific recommendations vary as to how to assess these risks, when to treat with antithrombotic therapy, and which treatments to use.

**Table 2:** Guidelines for the management of stroke in NVAF

	2012 AHA/ASA: Scientific Advisory [8]	2012 ACCP [11]	2012 ESC [7]	2014 AAN [9]^a^	2014 ACC/AHA/HRS [10]
Stroke risk	Guideline recommendations by stroke risk				
Low	CHADS_2_ = 0	CHADS_2_ = 0	CHA_2_DS_2_-VASc = 0	Clinicians might not offer anticoagulation to patients with NVAF who lack additional risk factors	CHA_2_DS_2_-VASc = 0
	Aspirin, based on patient preference, estimated bleeding risk if anticoagulated, and access to high-quality anticoagulation monitoring	No therapy suggested rather than antithrombotic therapy	No antithrombotic therapy recommended	Clinicians might offer antithrombotic therapy with aspirin or no therapy at all	Reasonable to omit antithrombotic therapy
		If antithrombotic therapy chosen, aspirin (75–325 mg/d) suggested rather than OAC or aspirin plus clopidogrel^b^			
Moderate	CHADS_2_ = 1	CHADS_2_ = 1	CHA_2_DS_2_-VASc = 1	Not discussed	CHA_2_DS_2_-VASc = 1
	Aspirin, based on patient preference, estimated bleeding risk if anticoagulated, and access to high-quality anticoagulation monitoring or adjusted-dose warfarin in appropriate patients	OAC suggested rather than no therapy. OAC suggested rather than aspirin alone or aspirin plus clopidogrel.^b^ If OAC unsuitable or not desired, aspirin plus clopidogrel^b^ suggested rather than aspirin alone	OAC therapy with adjusted-dose VKA (INR 2.0–3.0); a direct thrombin inhibitor (dabigatran); an oral factor Xa inhibitor (e.g., rivaroxaban, apixaban) should be considered, based upon an assessment of the risk of bleeding complications and patient preferences. For female patients aged <65 years with lone AF (CHA_2_DS_2_-VASc = 1 due to sex), no antithrombotic therapy should be considered		No therapy or OAC or aspirin may be considered
High	CHADS_2_≥2	CHADS_2_≥2	CHA_2_DS_2_-VASc ≥2		CHA_2_DS_2_-VASc ≥2
	Adjusted-dose warfarin in appropriate patients (in patients unsuitable for warfarin, aspirin plus clopidogrel^b^ offers more protection against stroke than aspirin but with an increased risk of major bleeding)	OAC suggested rather than no therapy, aspirin alone, or aspirin plus clopidogrel.^b^ If OAC unsuitable or not desired, aspirin plus clopidogrel^b^ suggested rather than aspirin alone	OAC therapy with adjusted-dose VKA (INR 2.0–3.0); a direct thrombin inhibitor (dabigatran); an oral factor Xa inhibitor (e.g., rivaroxaban, apixaban) recommended, unless contraindicated	Clinicians should routinely offer anticoagulation to patients with NVAF and a history of TIA or stroke	OAC recommended (warfarin, dabigatran, rivaroxaban, or apixaban)
Treatment options	Guideline recommendations by agent				
Adjusted-dose VKA	See recommendations by CHADS_2_ score	Patients with AF and mitral stenosis		INR of 2.0–3.0 likely reduces frequency and severity of ischemic stroke vs lower INR levels	Patients with mechanical heart valve (target INR 2.0–3.0 or 2.5–3.5 based on type and location of prosthesis)
		Patients with AF and stable CAD			Patients with NVAF and CHAD_2_DS_2_-VASc ≥2 with end-stage CKD (CrCl <15 mL/min) or on hemodialysis
		Patients with AF and ACS not undergoing stent placement (in combination with single antiplatelet for first 1–12 mo, after which treat as for patients with AF and stable CAD)			
Dabigatran	150 mg bid is an efficacious alternative to warfarin in patients with NVAF who have ≥1 additional risk factor for stroke and CrCl >30 mL/min		Recommended over adjusted-dose VKA in cases where OAC recommended	Probably more effective than warfarin for reducing risk of stroke or SE	Recommended for patients unable to maintain a therapeutic INR level with warfarin
	Reduce dosage to 75 mg bid in patients with moderate renal impairment (CrCl 15–30 mL/min)^c^		Dabigatran 150 mg bid recommended for most patients	Hemorrhage risk was similar overall between dabigatran 150 mg and warfarin; ICH was less frequent with dabigatran 150 mg than warfarin; GI bleeding more frequent with dabigatran 150 mg than with warfarin	May be considered in patients with renal impairment: 150 mg bid in patients with mild renal impairment (CrCl >30 mL/min); 150 mg or 75 mg bid in patients with moderate renal impairment (CrCl >30 mL/min); 75 mg bid in patients with severe renal impairment (CrCl 15–30 mL/min)
			• Dabigatran 110 mg bid recommended for:		
			• Elderly patients (aged ≥80 y)		
			• Concomitant use of interacting drugs		
			• HAS-BLED ≥3		
			• Moderate renal impairment (CrCl 30–49 mL/min)		
	Not recommended in patients with severe renal impairment (CrCl <15 mL/min)	Recommended over adjusted-dose VKA in cases where OAC recommended	Not recommended in patients with severe renal impairment (CrCl <30 mL/min)		Not recommended for patients with CrCl<15 mL/min
Rivaroxaban^d^	20 mg/d is a reasonable alternative to warfarin in patients with NVAF at moderate to high risk of stroke (prior history of TIA, stroke, or SE, or ≥2 additional risk factors) 15 mg/d may be considered in patients with renal impairment (CrCl 15–50 mL/min)^c^		Recommended over adjusted-dose VKA in cases where OAC recommended	In patients with NVAF at high risk of cerebral or systemic embolism. Probably as effective as warfarin for prevention of cerebral and systemic embolism, with no difference in risk of major bleeding episodes except GI bleeding	Recommended for patients unable to maintain a therapeutic INR level with warfarin
					May be considered in patients with renal impairment: 20 mg/d for patients with mild renal impairment (CrCl >50 mL/min); 15 mg/d for patients with moderate or severe renal impairment (CrCl 15–50 mL/min)
			Rivaroxaban 20 mg/d recommended for most patients		
			Rivaroxaban 15 mg/d recommended for:		
			• HAS-BLED ≥3		
			• Moderate renal impairment (CrCl 30–49 mL/min)		
	Should not be used in patients with severe renal impairment (CrCl <15 mL/min)	Not approved at time of guideline preparation	Not recommended in patients with severe renal impairment (CrCl <30 mL/min)	Associated with lesser frequency of ICH and fatal bleeding compared with warfarin	Not recommended for patients with CrCl < 15 mL/min
Apixaban^e^	As an alternative to warfarin or aspirin: 5 mg bid is relatively safe and efficacious in patients with NVAF who have ≥1 additional risk factor and ≤1 of the following additional criteria: age ≥80 y, weight ≤60 kg, or serum creatinine ≥1.5 mg/dL		Recommended over adjusted-dose VKA in cases where OAC recommended apixaban 5 mg bid	In patients with NVAF at moderate risk of embolism, 5 mg bid is likely more effective than warfarin	Recommended for patients unable to maintain a therapeutic INR level with warfarin
	• 2.5 mg bid may be considered in patients with ≥2 of the additional criteria described above^c^		Apixaban 2.5 mg bid recommended for patients with renal impairment	Superiority is related to decreased risk of bleeding and reduced mortality, while its effect on reduction in risk of cerebral and systemic embolism is not superior to warfarin	5.0 mg bid in patients with mild or moderate renal impairment or 2.5 mg bid in patients who meet dose reduction criteria (CrCl ≥1.5 mg/dL, ≥80 years of age, body weight ≤60 kg)
	Should not be used in patients with severe renal impairment (CrCl <25 mL/min)	Not approved at time of guideline preparation	Not recommended in patients with severe renal impairment (CrCl <30 mL/min)	Likely more effective than aspirin for decreasing risk of stroke or SE in patients with NVAF who have moderate risk of embolism and are not candidates for warfarin	No recommendation in patients with severe renal impairment or end-stage CKD
Edoxaban	Not approved at time of guideline preparation	Not approved at time of guideline preparation	Not approved at time of guideline preparation	Not approved at time of guideline preparation	Not approved at time of guideline preparation
Other agents				Oral anticoagulation is likely more effective than clopidogrel plus aspirin, but ICH is more common	
				Triflusal plus acenocoumarol and moderate-intensity anticoagulation (INR 1.25–2.0) is likely more effective than treatment with acenocoumarol alone and conventional-intensity anticoagulation	
				Combination of low-dose aspirin and dose-adjusted VKA therapy probably increases risk of hemorrhage	
				Combination of clopidogrel and aspirin reduces risk of major vascular events but increases risk of major hemorrhage compared with aspirin alone	

*AAN* American Academy of Neurology, *ACC* American College of Cardiology, *ACCP* American College of Chest Physicians, *ACS* acute coronary syndrome, *AF* atrial fibrillation, *AHA* American Heart Association, *ASA* American Stroke Association, *bid* twice daily, *CAD* coronary artery disease, *CHADS*_*2*_ Congestive heart failure, Hypertension, Age ≥65 y, Diabetes, Stroke or transient ischemic attack (doubled), *CHA*_*2*_ *DS*_*2*_ *-VASc* Congestive heart failure, Hypertension, Age ≥75 y (doubled), Diabetes, Stroke or transient ischemic attack (doubled), Vascular disease, Age 65–74 y, Sex category (female), *CKD* chronic kidney disease, *CrCl* creatinine clearance, *ESC* European Society of Cardiology, *GI* gastrointestinal, *HAS-BLED* Hypertension, Abnormal renal/liver function, Stroke, Bleeding history or predisposition, Labile INR, Elderly, Drugs/alcohol concomitantly, *HRS* Heart Rhythm Society, *ICH* intracranial hemorrhage, *INR* international normalized ratio, *NVAF* nonvalvular atrial fibrillation, *OAC* oral anticoagulant, *SE* systemic embolism, *TIA* transient ischemic attack, *VKA* vitamin K antagonist

^a^AAN recommends that clinicians use risk stratification tools to help determine stroke risk in patients with NVAF, but cautions physicians not to rigidly interpret anticoagulation thresholds suggested by these tools and does not stratify recommendations using a scoring system

^b^In the United States, clopidogrel and the more recently developed antiplatelet agents, prasugrel and ticagrelor, are used in patients with ACS, but none are indicated for stroke prevention in AF

^c^Recommendations made; however, safety and efficacy have not been established

^d^Rivaroxaban should be administered once daily with the evening meal

^e^2.5 mg bid if any 2 patient characteristics present: CrCl ≥1.5 mg/dL, ≥80 years of age, body weight ≤60 kg

The CHADS_2_ and CHA_2_DS_2_-VASc risk indexes predict the risk of stroke in patients with AF. Both the ACC/AHA/HRS guidelines and the ESC guidelines mention the ATRIA and HEMORR_2_ HAGES scores for bleeding risk, but acknowledge that the HAS-BLED risk index is most predictive of bleeding events. Together, these scoring systems can be used to evaluate an approach to treatment in patients with AF. The CHADS_2_ score is based on a patient’s history of Congestive heart failure (1 point), Hypertension (1 point), Age ≥75 years (1 point), Diabetes (1 point), and history of Stroke or transient ischemic attack (TIA, 2 points; Table 3) [33]. Scores of 0, 1, or ≥2 indicate low, moderate, or high stroke risk, respectively [8, 34]. The CHADS_2_ risk scoring index has been thoroughly validated [33–35]; is simple, inexpensive, and broadly applicable; and most importantly, it allows identification of patients at low risk of stroke who may not receive significant benefit from anticoagulant therapy. However, the CHADS_2_ risk score has limitations, primarily that patients deemed at low risk with CHADS_2_ still have a stroke risk of 2.2 % per year, in addition, there is a lack of differentiation between stroke risk factors and risk factors that do not consistently predict stroke (i.e., congestive heart failure). As a result, some patients may be identified as moderate- rather than low-risk stroke candidates, or vice versa [33–35]. Also, in keeping the CHADS_2_ risk score simple, some common stroke risk factors are not included [35, 36].

**Table 3:** Stroke risk scoring systems: CHADS_2_ and CHA_2_DS_2_-VASc

CHADS_2_ score					
Scoring system			Stroke rates associated with CHADS_2_ score		
	Risk factor	Points	Risk score	Adjusted stroke rate (%/y)^a, b^ (95 % CI)	Risk of stroke
C	CHF	1	0	1.9 (1.2, 3.0)	Low
H	Hypertension	1	1	2.8 (2.0, 3.8)	Moderate
A	Age ≥75 y	1	2	4.0 (3.1, 5.1)	
D	Diabetes	1	3	5.9 (4.6, 7.3)	
S_2_	Prior stroke/TIA	2	4	8.5 (6.3, 11.1)	High
			5	12.5 (8.2, 17.5)	
			6	18.2 (10.5, 27.4)	
CHA_2_DS_2_-VASc score					
Scoring system			Stroke rates associated with CHA_2_DS_s_-VASc score		
	Risk factor	Points	Risk score	TE rate during 1 year (% [95 % CI])^c, d^	Risk of stroke
C	CHF/LV dysfunction	1	0	0	Low
H	Hypertension	1	1	0.6 (0.0, 3.4)	Moderate
A_2_	Age ≥75 y	2	2	1.6 (0.3, 4.7)	
D	Diabetes	1	3	3.9 (1.7, 7.6)	High
S_2_	Prior stroke/TIA/TE	2	4	1.9 (0.5, 4.9)	
V	Vascular disease (prior MI, PAD, or aortic plaque)	1	5	3.2 (0.7, 9.0)	
A	Age 65–74 y	1	6	3.6 (0.4, 12.3)	
Sc	Sex category (female sex)	1	7	8.0 (1.0, 26.0)	

*CHF* congestive heart failure, *CI* confidence interval, *LV* left ventricular, *MI* myocardial infarction, *PAD* peripheral artery disease, *TE* thromboembolism, *TIA* transient ischemic attack

^a^Adapted from Gage BF et al. JAMA. 2001;285:2864–70 [33]

^b^Adjusted stroke is expected stroke rate per 100 person-years from exponential survival model, assuming no aspirin was taken

^c^Adapted from Lip GYH et al. Chest. 2010;137:263–72 [34]

^d^p value for trend = 0.003

The ACC/AHA/HRS and ESC advocate using the CHA_2_DS_2_-VASc score instead of the CHADS_2_ score to assess stroke risk. The ESC states that the CHA_2_DS_2_-VASc score more accurately identifies “truly low-risk” patients with AF who are not likely to benefit from oral anticoagulant therapy (Table 3) and, conversely, those patients who may benefit from anticoagulation because they are at risk for stroke or systemic thromboembolism [7]. The CHA_2_DS_2_-VASc score has been shown to identify some patients considered low risk by CHADS_2_ (score of 0) to actually be at a moderate risk of stroke [37–39]. The CHA_2_DS_2_-VASc considers additional risk factors, including Vascular disease (1 point) and Sex category (1 score point for female sex), and heightens the risk rendered by older age, assigning 2 points rather than 1 (as with CHADS_2_) for age ≥75 years, as well as 1 point for the risk factor of age 65–74 years [34]. With CHA_2_DS_2_-VASc, patients with scores of 0, 1, or ≥2 are considered to be at low, moderate, or high risk of stroke, respectively [34]. In one study of the predictive value of risk classification schemes, when patients were categorized by CHA_2_DS_2_-VASc score, 0 % of low-risk patients, 0.6 % of moderate-risk patients, and 3 % of high-risk patients experienced a thromboembolic event. In contrast, when the same cohort of patients was classified according to the CHADS_2_ scoring system, 1.4 % of low-risk, 1.9 % of moderate-risk, and 3.1 % of high-risk patients experienced an event [34]. Despite these potential advantages, the CHA_2_DS_2_-VASc score index lacks the extensive validation that CHADS_2_ has received, and studies have demonstrated a similar predictive ability for the 2 indexes [11, 34, 40].

The AAN guidelines explicitly acknowledge the discrepancies between the risk scoring systems, stating “although multiple risk stratification tools are available for estimating the absolute stroke risk of patients with NVAF, the absolute stroke risks estimated by these tools vary widely [9].” Rather than a score-based recommendation, the AAN acknowledges that determining when the benefit of reducing stroke risk will outweigh the harm of increased bleeding risk is difficult, and emphasizes the importance of patient preference and physician judgment under these circumstances [9].

Concurrent assessment of stroke and bleeding risk presents a clinical challenge, as many of the risk factors for stroke and bleeding overlap [11]. Indeed, a subgroup analysis of the RE-LY trial demonstrated an increased risk for bleeding associated with higher CHADS_2_ scores in patients receiving oral anticoagulation [41]. Among the bleeding risk scores available, HAS-BLED has been independently validated, and correlates well with intracranial hemorrhage risk (Table 4) [7, 42]. HAS-BLED includes the following risk factors: Hypertension, Abnormal renal/liver function, Stroke, Bleeding history or predisposition, Labile international normalized ratio (INR), Elderly (age ≥65 years), and Drugs (concomitant antiplatelet/nonsteroidal anti-inflammatory drug or alcohol) [7, 11]. While the ESC suggests using the HAS-BLED bleeding risk score [7], the ACC/AHA/HRS, ASA, and ACCP do not endorse a specific scoring system, but do advise that an assessment of bleeding risk is needed [8, 11, 41]. The ESC guidelines recommend caution along with efforts to correct potentially reversible bleeding risk factors when prescribing antithrombotic therapy for patients with a HAS-BLED score ≥3. Importantly, the ESC guidelines emphasize that: *whereas the HAS-BLED score should be used to identify modifiable bleeding risks, a high HAS-BLED score alone should not exclude patients from oral anticoagulant treatment* [7].

**Table 4:** Bleeding risk scoring system: HAS-BLED. Adapted from Pisters R et al. Chest. 2010;138;1093–100 [42]

	Risk factor	Points
H	Hypertension	1
A	Abnormal renal and liver function (1 point each)	1 or 2
S	Stroke	1
B	Bleeding	1
L	Labile INRs	1
E	Elderly (age >65 y)	1
D	Drugs or alcohol (1 point each)	1 or 2
Risk score	Bleeds per 100 patient-years	Risk of bleeding
0	1.13	Low
1	1.02	Moderate
2	1.88	
3	3.74	High
4	8.70	
5	12.50	

*INR* international normalized ratio

NOACs have been included in the most recent updates to guidelines for stroke prevention in patients with AF (Table 2) [7–11]. It should be noted that edoxaban has not been included in the guideline recommendations because it was not approved at the time of publication. The ESC recommends the NOACs over warfarin based on their better efficacy, safety, and convenience [7], whereas the AHA/ASA recommend the NOACs as an alternative to warfarin in the presence of at least 1 additional risk factor for stroke [8]; neither guideline recommends one NOAC over another. While the ACC/AHA/HRS recommend any of the 3 NOACs (i.e., dabigatran, rivaroxaban, or apixaban) as an efficacious alternative to warfarin [10], the ACCP guidelines recommend dabigatran 150 mg twice daily in place of adjusted-dose VKA [11]. (The ACCP guidelines include dabigatran but not apixaban or rivaroxaban in their recommendations, as the latter 2 NOACs were not yet approved when the guidelines were drafted [11].) The ACCP guidelines favor oral anticoagulants over aspirin alone or, for patients at intermediate to high risk of stroke for whom warfarin is unsuitable, suggest aspirin given with clopidogrel; where oral anticoagulation is indicated, dabigatran is recommended over warfarin [11]. The AAN guideline update offers recommendations for specific NOACs in lieu of or as an alternative to warfarin based on type of stroke risk (i.e., general risk [dabigatran 150 mg], high risk of cerebral or systemic embolism [SE] [rivaroxaban], moderate risk of embolism [apixaban 5 mg twice daily]) [9]. Additionally, the AAN update recommends apixaban 5 mg bid over aspirin for reducing risk of stroke or SE [9], and the AAN guidelines include a recommendation for the addition of clopidogrel to aspirin as an alternative for patients with AF in whom warfarin is considered unsuitable [9]. It should be noted that while the ACC, ACCP, AHA, ASA, HRS, and ESC use the CHADS_2_, CHA_2_DS_2_-VASc, and/or HAS-BLED risk scores to determine their treatment recommendations, these risk scoring systems are based on the results of trials during the era in which patients were receiving warfarin, aspirin, or placebo, and not the new NOACs [10, 43–45]. The study that validated the CHADS_2_ score measured stroke risk among patients who were not receiving any form of anticoagulant therapy [33].

Performance and quality measures from the Joint Commission, a US healthcare accrediting organization formerly known as the Joint Commission on Accreditation of Healthcare Organizations, and from the ACC/AHA also provide guidance on the treatment of patients with NVAF [46, 47]. Published in 2008, the Joint Commission’s *Stroke Performance Implementation Guide* recommends warfarin (unless contraindicated) for patients with AF-related stroke [47]. ACC/AHA performance measures, also published in 2008, recommend antithrombotic therapy with aspirin or warfarin for patients with NVAF based on the patient’s stroke risk category [46].

More recently, the Stroke and Stroke Rehabilitation Work Group provided guidance in 2012 with quality measures to improve outcomes in stroke, TIA, and stroke rehabilitation. Composed of the AAN, American College of Radiology, National Committee for Quality Assurance, and the American Medical Association–convened Physician Consortium for Performance Improvement® (PCPI®), the Stroke and Stroke Rehabilitation Work Group advocates anticoagulant therapy with dabigatran, rivaroxaban, warfarin, or low-molecular-weight heparin at discharge for patients with AF [48].

### Application of treatment guidelines to clinical practice

With multiple oral anticoagulants now available to reduce the risk of stroke in patients with NVAF, guidelines and treatment paradigms will continue to evolve to reflect findings from clinical trials and emerging clinical experience. The hypothetical cases that follow explore how clinicians might apply guidelines and clinical trial data to treatment decisions for the patient throughout the clinical course of NVAF.

### Hypothetical patient case 1: Balancing the benefit of thromboprophylaxis with the risk of bleeding

BL is a 64-year-old white woman who presents to the emergency room with cellulitis of the left forearm that has not responded to outpatient antibiotic treatment. BL takes metoprolol for hypertension, but no other medications. She has no history of diabetes. She is a nonsmoker who exercises regularly and occasionally has a glass of wine with dinner. Physical examination reveals the following: body mass index (BMI), 22 kg/m^2^ (height, 5′7′′; weight, 139 lbs); blood pressure, 144/82 mm Hg; pulse, 78 bpm, irregular; temperature, 101 °F. On electrocardiogram (ECG), an irregularly irregular rhythm is noted with no ST-elevation and a normal axis. Apart from moderate cellulitis of the left forearm and associated signs of infection, no other abnormal findings are noted during the physical examination.

BL is admitted to the hospital and the cellulitis responds to intravenous antibiotics. She asks if her irregular heart rhythm will require additional treatment or a repeat of treatment she had taken for it previously. Upon further inquiry, her hospitalist notes that 2 years ago BL had been examined for complaints of breathlessness and a rapid heart rate. After a complete cardiac workup at that time, she had been diagnosed with paroxysmal NVAF and was prescribed warfarin. BL states she didn’t like the frequent blood tests that were associated with warfarin and often found it difficult to get to the anticoagulant clinic due to her work schedule. During a follow-up visit approximately 2 months after BL had started taking warfarin, her physician found that she was still in AF, although her heart rate had returned to normal, and although he recommended that she continue taking warfarin, she chose to stop it at that time.

#### Case discussion

Due to her history of hypertension, BL has a HAS-BLED score of 1, a CHADS_2_ score of 1, and a CHA_2_DS_2_-VASc score of 2. Her HAS-BLED score indicates a low risk of bleeding. Her CHADS_2_ score indicates a moderate risk of stroke, which is associated with a stroke rate of 2.8 % per year [33]. In contrast, her CHA_2_DS_2_-VASc score places her in the high-risk category, although the rate of stroke associated with a CHA_2_DS_2_-VASc score of 2 is 2.2 % per year, similar to that associated with her CHADS_2_ score [10]. Her moderate to high stroke risk (depending on the scoring system used) suggests the importance of stroke prevention in this patient.

Treatment guidelines provide various recommendations for patients with a CHADS_2_ score of 1 (or a CHA_2_DS_2_-VASc score of ≥2), ranging from daily aspirin to oral anticoagulation with warfarin or a NOAC (Table 2) [7–11]. In the present case, however, an effective treatment choice for BL must also consider her low risk of bleeding and her past noncompliance with warfarin therapy. Based on BL’s history of difficulty maintaining a therapeutic INR while on warfarin and adhering to monitoring requirements, warfarin would not be the ideal first choice for this patient. Instead, one of the NOACs may provide a safe, efficacious, and convenient alternative.

Consideration of pharmacologic properties and patient-specific characteristics, such as comorbidities, may help in the identification of a specific NOAC for stroke prevention (Table 5) [23–31, 49–58]. However, in this case, BL’s medical history and concomitant medication (metoprolol) do not rule out any of the NOACs. Given a CHADS_2_ score of 1, one might consider aspirin versus a NOAC. In contrast, her CHA_2_DS_2_-VASc score of 2 would suggest that an anticoagulant is a more appropriate choice. As noted previously, aspirin is less effective than warfarin in preventing stroke in patients with NVAF [21]. Of the NOACs, only apixaban has been studied head-to-head versus aspirin (AVERROES trial) [32]. The AVERROES trial demonstrated greater efficacy with apixaban in significantly reducing the risk of stroke and SE compared with aspirin (hazard ratio 0.45; 95 % confidence interval, 0.32, 0.62; *p* < 0.001; Table 1) [32]. For example, the annual rate of stroke or SE was 1.6 % (51/2808) with apixaban versus 3.7 % (113/2791) with aspirin, with no significant difference between treatments in rates of major (*p* = 0.57), gastrointestinal (GI; *p* = 0.71), or intracranial (*p* = 0.69) bleeding [31]. Based on the results of the AVERROES trial, and given the low risk of bleeding anticipated for BL based on her HAS-BLED score of 1, using an oral anticoagulant instead of aspirin on balance appears to be the better choice, considering that complications of stroke can be life-debilitating.

**Table 5:** Clinical and pharmacologic properties of apixaban, rivaroxaban, dabigatran, and edoxaban

Criteria	Dabigatran [28]	Rivaroxaban [30]	Apixaban [29]	Edoxaban [27]
Anemia	Contraindicated	Contraindicated in patients with hemoglobin <10 g/dL	Contraindicated in patients with hemoglobin <9 g/dL	NA
Bleeding risk	Contraindicated in patients with hemoglobin <10 g/dL and patients with active pathologic bleeding			
	Can cause serious and sometimes fatal bleeding			
	Concomitant drugs affecting hemostasis can increase bleeding risk, including platelet aggregation inhibitors, heparin, fibrinolytic therapy, and chronic use of NSAIDs	Concomitant drugs affecting hemostasis can increase bleeding risk, including NSAIDs, heparin, aspirin, platelet aggregation inhibitors, other antithrombotic drugs, and fibrinolytic therapy	Concomitant drugs affecting hemostasis can increase bleeding risk, including platelet aggregation inhibitors, other antithrombotic drugs, heparin, thrombolytic agents, SSRIs, SNRIs, and chronic use of NSAIDs	Concomitant use of drugs affecting hemostasis may increase the risk of bleeding, including aspirin and other antiplatelet agents, other antithrombotic agents, fibrinolytic therapy, and chronic use of NSAIDs
Interruption for surgery/procedures	To reduce risk of bleeding, discontinue dabigatran 1–2 d (CrCl ≥50 mL/min) or 3–5 d (CrCl <50 mL/min) before invasive or surgical procedures	To reduce risk of bleeding, discontinue rivaroxaban at least 24 h prior to surgical or other invasive procedures	To reduce risk of bleeding, discontinue apixaban at least 48 h prior to elective surgery or invasive procedures with a moderate or high risk of unacceptable or clinically significant bleeding, and at least 24 h prior to elective surgery or invasive procedures with a low risk of bleeding or where the bleeding would be noncritical in location and easily controlled	To reduce the risk of bleeding, discontinue edoxaban at least 24 hours before invasive or surgical procedure
Drug interactions	Avoid concomitant use with P-gp inducers (e.g., rifampin)	Avoid concomitant use with combined P-gp and strong CYP3A4 inhibitors (e.g., ketoconazole, ritonavir) or inducers (carbamazepine, phenytoin, rifampin, St. John’s wort)	Reduce apixaban dosage to 2.5 mg bid or avoid concomitant use with strong dual inhibitors of CYP3A4 and P-gp (e.g., ketoconazole, itraconazole, ritonavir, or clarithromycin)	Co-administration with anticoagulants, antiplatelet drugs and thrombolytics may increase the risk of bleeding
	For patients with moderate renal impairment (CrCl 30–50 mL/min), dabigatran dose may be reduced to 75 mg bid when administered concomitantly with the P-gp inhibitor dronedarone or systemic ketoconazole. Dose adjustment not required with P-gp inhibitors (verapamil, amiodarone, quinidine, and clarithromycin)	For patients with CrCl 15–50 mL/min, rivaroxaban may be used concomitantly with combined P-gp and weak or moderate CYP3A4 inhibitors (e.g., amiodarone, diltiazem, verapamil, quinidine, ranolazine, dronedarone, felodipine, erythromycin, and azithromycin) *only* if the potential benefit justifies the potential risk	Avoid concomitant use with strong dual inducers of CYP3A4 and P-gp (e.g., rifampin, carbamazepine, phenytoin, St. John’s wort) because such drugs will decrease exposure to apixaban	Avoid concomitant use with rifampin
	Avoid concomitant use with P-gp inhibitors in patients with severe renal impairment (CrCl 15–30 mL/min)			No dose reduction is recommended in patients taking concomitant P-gp inhibitors
Hepatic impairment	Large intersubject variability but no evident consistent change in exposure or PD in patients with moderate hepatic impairment	Avoid use in moderate and severe hepatic impairment or with any hepatic disease associated with coagulopathy	No dose adjustment necessary in patients with mild hepatic impairment	Use of edoxaban in patients with moderate or severe hepatic impairment is not recommended as these patients may have intrinsic coagulation abnormalities. No dose reduction is required in patients with mild hepatic impairment
			No dosing recommendations available for patients with moderate hepatic impairment because of lack of clinical experience with apixaban in these patients	
			Not recommended in patients with severe hepatic impairment	
Renal elimination	80 % [69]	36 % [68]	27 % [29, 66]	50 % [57]
Renal impairment	Contraindicated in patients with CrCl <15 mL/min; reduce dosage to 75 mg bid if CrCl 15–30 mL/min	Avoid use in patients with CrCl <15 mL/min	Reduce dosage to 2.5 mg bid in patients with serum creatinine ≥1.5 mg/dL and either age ≥80 years or body weight ≤60 kg	Reduce edoxaban dose to 30 mg qd in patients with CrCl 15-50 mL/min. Not recommended in patients with CrCl<15 mL/min
			5 mg bid in patients with ESRD maintained on hemodialysis. Reduce dose to 2.5 mg bid in patients with ESRD aged ≥80 years or body weight ≤60 kg	
Reversal antidote	In progress; none approved for use	In progress; none approved for use	In progress; none approved for use	In progress; none approved for use
Tested reversal strategies^a^	Dialysis [54]	PCC (Cofact) [50]	rFVIIa [51]	rFVIIa [56]
	aPCC (FEIBA®) [55]	aPCC (FEIBA®) [53]	aPCC (FEIBA®) [51]	aPCC (FEIBA®) [56]
	PER977 (when available) [76]	Andexanet alfa (when available) [52]	Activated charcoal [71]	PPSB-HT [56]
		PER977 (when available) [76]	Andexanet alfa (when available) [49, 52]	
			PER977 (when available) [76]	

*aPCC* activated prothrombin complex concentrate; *bid* twice dailym, *CrCl* creatinine clearance, *CYP3A4* cytochrome P450 3A4, *ESRD* end-stage renal disease, *FEIBA*® factor VIII inhibitor bypass activity, *NA* not available, *NSAID* nonsteroidal anti-inflammatory drug, *PCC* prothrombin complex concentrate, *PD* pharmacodynamics, *P-gp* P-glycoprotein, *PPSB-HT* prothrombin complex concentrate, *rFVIIa* recombinant activated factor VIIa, *SNRI* serotonin/norepinephrine reuptake inhibitor, *SSRI* selective serotonin reuptake inhibitor

### Hypothetical patient case 2: Identifying and managing stroke risk in the midst of comorbidities and a high risk of bleeding

AS is a 73-year-old black man with type 2 diabetes, hypertension, and persistent NVAF who presents to the emergency room with complaints of occasional palpitations, lightheadedness, and a productive cough. He takes metformin for diabetes, valsartan with chlorthalidone for hypertension, and a daily aspirin tablet for his AF. He is a nonsmoker and leads a generally sedentary lifestyle. On physical examination, AS has a BMI of 34 kg/m^2^ (height 5′10′′; weight, 237 lbs); blood pressure, 154/90 mm Hg; pulse, 100 bpm; and temperature, 102 °F. His lab and test results were consistent with a diagnosis of acute pneumonia.

AS is admitted to the hospital and treated for pneumonia. Past history reveals that he was diagnosed 2 years ago with AF and treated at that time with warfarin, which was stopped 1 year ago due to a GI bleed. Subsequently, he was put on an aspirin regimen of 75 mg per day. During the current visit AS expresses concern to the hospitalist about his persistent AF, diabetes, and high blood pressure, all of which increase his risk of stroke. He says he wants to resume oral anticoagulant treatment, which he thinks may be better for him than aspirin.

#### Case discussion

This patient’s age and history of hypertension and diabetes give him a CHADS_2_ score of 2 (and CHA_2_DS_2_-VASc score of 3), which indicates an increased risk of stroke and is associated with a stroke rate of 4.0 % per year based on his CHADS_2_ score, or 3.2 % per year based on his CHA_2_DS_2_-VASc score [10, 33]. His age (≥65 years), past history of GI bleeding, and hypertension add up to a HAS-BLED score of 3, which is linked to an increased risk of bleeding, and data show it to be associated with a bleeding rate of 3.74 % per year [42]. The high risk of both stroke and bleeding call for immediate stroke prevention. Guideline recommendations for patients with a CHADS_2_ score of 2 (or a CHA_2_DS_2_-VASc score ≥2) include oral anticoagulation with warfarin or a NOAC (Table 2) [7, 8, 10, 11]. AS has a history of GI bleeding with warfarin, and has several characteristics associated with poor INR control including his age, non-white race, and diabetes [59–61]. A NOAC may provide a safer alternative to warfarin. However, in the RE-LY and ROCKET-AF trials, dabigatran and rivaroxaban, respectively, were associated with similar rates of major bleeding and increased rates of GI bleeding versus warfarin [23, 25]. In the RE-LY trial, the rates of major bleeding for dabigatran 110 mg and 150 mg bid were 2.71 % per year (*p* = 0.003) and 3.11 % per year (*p* = 0.31), respectively, compared with 3.36 % per year with warfarin [23]. Rates of GI bleeding with dabigatran 110 mg and 150 mg versus warfarin were 1.12 % per year (*p* = 0.43) and 1.50 % per year (*p* < 0.001) versus 1.02 % per year, respectively [23]. Multiple GI bleeding events with dabigatran have also been reported in recent postmarketing surveillance [62]. In the ROCKET-AF trial, the rates of major bleeding were 3.6 % per year and 3.4 % per year for rivaroxaban and warfarin, respectively (*p* = 0.58), and GI bleeding occurred in 3.2 % of patients with rivaroxaban versus 2.2 % of patients with warfarin (*p* < 0.001) [25]. In the ARISTOTLE trial comparing apixaban with warfarin, apixaban demonstrated a 31 % reduction in major bleeding and no increased risk of GI bleeding compared with warfarin [24]. Non-bleeding GI adverse events (AEs) were more common with dabigatran than with rivaroxaban in the RE-LY and ROCKET-AF trials, respectively, versus warfarin (16.9 % vs. 9.4 %; 5.33 % vs. 5.57 %, respectively) [25, 63]. In the RE-LY trial, dyspepsia was the most common AE among patients receiving dabigatran, occurring significantly more often with either dose of dabigatran than with warfarin (*p* < 0.001 for both doses) [25]. An analysis of data from the RE-LY trial found that non-bleeding upper GI AEs were generally mild or moderate with dabigatran and warfarin [63]. However, patients receiving dabigatran compared with warfarin were more likely to discontinue the study drug due to non-bleeding upper GI AEs or dyspepsia-like symptoms (4.0 % vs. 1.7 %; *p* < 0.001, respectively) [63]. Similar findings were not observed for apixaban or rivaroxaban in the ARISTOTLE and ROCKET-AF trials, respectively [24, 25]. In the ROCKET-AF trial, the only difference in incidence of AEs between treatment groups was epistaxis, which occurred more frequently in patients receiving rivaroxaban than patients receiving warfarin (10.14 % vs. 8.55 %, *p* < 0.05) [25]. In the ARISTOTLE trial, the incidence of AEs was similar in the treatment groups [24].

AS’s comorbidities and concomitant medications should also be considered when selecting a NOAC. Having diabetes places AS at increased risk of bleeding when taking oral anticoagulants, and also poses the possibility of renal impairment [28, 64, 65]. Of the 3 NOACs, apixaban has the least renal elimination (~27 %) compared with rivaroxaban (~36 %) and dabigatran (~80 %), and is also eliminated via biliary and possibly direct intestinal excretion (Table 5) [27–29, 64–71]. Apixaban also demonstrated superiority over warfarin in reducing risk of stroke/SE, major bleeding, and mortality, irrespective of renal function [72]. Apixaban 5 mg twice daily may be a good NOAC option for this patient who is at high risk of bleeding and with comorbidities [7, 8, 10].

## Discussion and conclusions

Conventional treatment options for reducing the risk of stroke in patients with NVAF, including VKAs and aspirin, have limitations that currently leave many patients undertreated or untreated, and thus, suboptimally protected from stroke. The NOACs provide an additional treatment pathway that may help address this trend by offering treatment options with equivalent or improved efficacy and safety compared with warfarin. Current treatment guidelines have begun to incorporate recommendations for use of the available NOACs, and as additional data emerge and guidelines are further updated, these recommendations will likely evolve. In the interim, clinicians may need to consider the recommendations of published guidelines while also weighing results of clinical trials on NOACs. Furthermore, real-world experience will provide clinicians with additional insight into their treatment decisions. The challenge, however, will be for clinicians to assess the benefits and risks of treatment within the framework of the individual characteristics that define each patient case scenario. Use of scoring systems to assess stroke and bleeding risk; consideration of patient characteristics such as concomitant medications, age, and renal failure; and an understanding of relevant pharmacologic characteristics of the NOACs will all assist in these treatment decisions (Table 5).

When comparing the results of clinical trials of each NOAC versus warfarin, subtle differences in clinical outcomes become evident, such as rates of stroke, SE, all-cause mortality, major bleeding, and intracranial hemorrhage. For example, compared with warfarin, dabigatran 150 mg provided greater risk reduction for stroke and SE in the RE-LY trial than did apixaban, rivaroxaban, or edoxaban in the ARISTOTLE, ROCKET-AF, and ENGAGE AF-TIMI 48 trials, respectively (Table 1) [23–28]. However, edoxaban and apixaban had a greater risk reduction for major bleeding versus warfarin than did dabigatran, as well as greater risk reduction for stroke and SE versus warfarin than did rivaroxaban. Apixaban and dabigatran 150 mg had a similar risk reduction for intracranial hemorrhage, but less than edoxaban 30 mg and dabigatran 110 mg and more than rivaroxaban. Rivaroxaban, apixaban, dabigatran 150 mg, and edoxaban provided comparable risk reduction versus warfarin for all-cause mortality [23–28]. Three formal indirect comparisons of the ARISTOTLE, RE-LY, and ROCKET-AF trials confirm these observations with regard to rivaroxaban, apixaban, and dabigatran [73–75]. One study quantified the benefits and risks of the NOACs with the following odds ratios (ORs) for stroke or SE: rivaroxaban versus dabigatran 150 mg, OR, 1.35 (*p* = 0.04); rivaroxaban versus dabigatran 110 mg, OR, 0.97 (*p* = 0.81); apixaban versus dabigatran 150 mg, OR, 1.22 (*p* = 0.18); apixaban versus dabigatran 110 mg, OR, 0.88 (*p* = 0.34); apixaban versus rivaroxaban, OR, 0.90 (*p* = 0.43). For major bleeding, the estimated ORs were: rivaroxaban versus dabigatran 150 mg, OR, 1.10 (*p* = 0.36); rivaroxaban versus dabigatran 110 mg, OR, 1.28 (*p* = 0.02); apixaban versus dabigatran 150 mg, OR, 0.74 (*p* = 0.004); apixaban versus dabigatran 110 mg, OR, 0.87 (*p* = 0.17); apixaban versus rivaroxaban, OR, 0.68 (*p* < 0.001) [74]. Indirect comparisons like these must be considered with caution because of differences in trial design and patient populations.

With the availability of the NOACs, success in reducing the risk of stroke among patients with NVAF no longer has to pivot on the suitability of warfarin for patients. Until treatment guidelines are updated to incorporate all available data on the NOACs, clinicians would be well advised to consider recent clinical trial data for these agents alongside current treatment guidelines for stroke prevention in AF. Such attentiveness may help render treatment decisions that accurately address the constellation of characteristics composing each individual case and result in safe, high-quality, and cost-effective patient care.

## Abbreviations

AEs: Adverse events
ACC: American College of Cardiology
ACCP: American College of Chest Physicians
AF: Atrial fibrillation
AHA: American Heart Association
AAN: American Academy of Neurology
AS: American Stroke Association
BMI: Body mass index
ESC: European Society of Cardiology
GI: Gastrointestinal
HRS: Heart Rhythm Society
NVAF: Nonvalvular AF
NOACs: Novel oral anticoagulants
OR: Odds ratio
PCPI^®^: Physician Consortium for Performance Improvement^®^
SE: Systemic embolism
TIA: Transient ischemic attack
VKA: Vitamin K antagonist
